# Catheter ablation in patients on mechanical circulatory supports for cardiogenic shock

**DOI:** 10.1371/journal.pone.0332597

**Published:** 2025-09-15

**Authors:** Milan Dusik, Stepan Havranek, Daniel Rob, Jan Simek, Martin Valek, Zdenka Fingrova, Tomas Boucek, Jan Pudil, Ales Linhart, Jan Belohlavek

**Affiliations:** 1 2nd Department of Medicine—Department of Cardiovascular Medicine, First Faculty of Medicine, Charles University in Prague and General University Hospital in Prague, Prague, Czech Republic; 2 Institute for Heart Diseases, Wroclaw Medical University, Wrocław, Poland; Azienda Ospedaliero Universitaria Careggi, ITALY

## Abstract

Short-term mechanical circulatory supports (MCS) are used to stabilize patients with severe cardiogenic shock (CS). Catheter ablation may be an option to suppress recurrent arrhythmias preventing MCS weaning. We retrospectively analysed a dedicated registry to identify CS patients who underwent a catheter ablation between January 2020 and August 2024 for treatment resistant and hemodynamically significant arrhythmias while being on the MCS. Patients with supraventricular and ventricular tachycardias (SVT/VT) were analysed separately. Nine patients (8 males, 69 [IQR 60;74] years) were ablated for a refractory VT. Impella CP was used in 6 patients, VA ECMO in 2 patients, and 1 patient was on ECPELLA. Seven patients (78%) were successfully weaned off the MCS after the catheter ablation. 3 patients (33%) died within 30 days. The arrhythmia recurred in 5 patients (56%). Significant complications of MCS were reported in 6 patients (66%). The catheter ablation was complicated in one patient. SVT ablation was performed in 4 patients (3 males, 73 [IQR 67; 78] years, 1x VA ECMO, 2x Impella CP, 1x Impella 5.5). Three patients with atrial fibrillation were treated by a non-selective AV node ablation (pace and ablate strategy). One patient underwent an ablation of focal atrial tachycardia. The MCS was successfully explanted in all patients and no patient died in 30 days. The MCS use was complicated in one patient. Catheter ablation of refractory arrhythmias in CS patients treated by MCS is a safe and feasible approach to facilitate the MCS weaning process.

## Introduction

Cardiogenic shock (CS) is an acute and life-threatening condition defined by a primary cardiac disorder resulting in low cardiac output, global tissue hypoperfusion, and organ dysfunction. Despite advances in treatment and the implementation of novel strategies, including mechanical circulatory supports (MCS), the prognosis of CS patients remains serious with 30-day mortality as high as 50% [1].

Heart rhythm disorders are common in CS patients. Arrhythmias can either represent a CS complication or can be the primary cause [2]. According to the available data, atrial fibrillation (AF) occurs in about 20% of CS cases and the incidence of ventricular fibrillation is reported to be 9–19% [3]. In 12% of patients, CS is triggered by sustained ventricular tachycardia (VT) [4]. Its presence leads to impaired ventricular contraction and diminished time for ventricular filling, both resulting in decreased cardiac output. Supraventricular arrhythmias (SVTs) are generally less dangerous. However, in patients with previous left ventricular dysfunction, even SVT can quickly lead to a deterioration [5]. It is important to remember that a persistent SVT can itself cause a left ventricle dysfunction known as tachycardia induced cardiomyopathy. When unrecognized and untreated, this can eventually present as an acute CS [6].

Immediate sinus rhythm restoration using electrical cardioversion is the first line treatment of arrhythmias in hemodynamically unstable patients, according to the guidelines. The management of recurrent or incessant arrhythmias is more challenging. Beside pharmacotherapy, catheter ablation is an established way of treatment for both supraventricular and ventricular arrhythmias [7–9].

**Fig 1:**
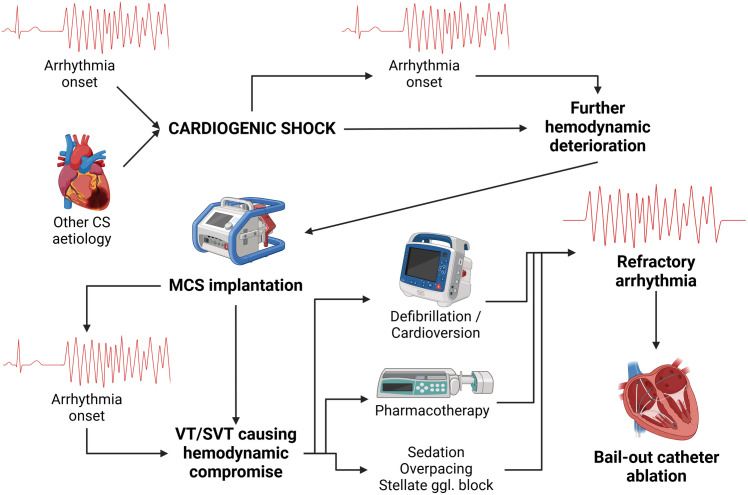
Arrhythmias in cardiogenic shock. Arrhythmias can be the primary cause of CS or they can appear later as a secondary complication. Bail-out catheter ablation in patients on MCS is an option that should be considered when the heart rhythm disorder is causing a significant hemodynamic compromise and is resistant to other treatment modalities. Abbreviations: CS – cardiogenic shock, ggl. – ganglion, MCS – mechanical circulatory support, SVT – supraventricular tachycardia, VT – ventricular tachycardia. *(Created in BioRender. Dusik, M. (2025)* https://BioRender.com/x36g205*).*

Short-term MCS including VA ECMO and Impella devices should be considered in selected patients to achieve hemodynamic stability until the underlying cause of the CS can be addressed and heart function recovers [10]. In patients with recurrent arrhythmias, MCS removal is usually not feasible until the heart rhythm is stabilized. When pharmacotherapy and cardioversion fail, performing a successful catheter ablation on MCS may be a crucial step for both the successful management and the patient’s outcome (Fig 1).

In this article, we provide a single-centre experience on the catheter ablation of treatment resistant supraventricular and ventricular arrhythmias in patients with cardiogenic shock treated by MCS.

## Methods

We thoroughly screened a dedicated registry of adult patients who underwent catheter ablation in the tertiary cardiac centre (General University Hospital in Prague, Czech Republic) between January 2020 and August 2024 to retrospectively identify all eligible patients for this analysis. The main inclusion criteria were: (1) CS treated by a short-term MCS (VA ECMO, Impella device or their combination), and (2) the presence of a refractory arrhythmia preventing the MCS weaning that was treated by a catheter ablation. Both patients with supraventricular and ventricular arrhythmias were included, but their data were analysed separately. Another registry of all patients treated by MCS was used to validate the collected data (e.g., the patient had to be included in both registries (Ablation registry, MCS registry)) to fit this analysis. Further details were obtained from the patients’ medical records.

For this study, we used a definition of CS which is routinely accepted in our institution, including: (1) blood pressure lower than 90 mmHg or the need for catecholamines, (2) clinical signs of end-organ hypoperfusion, supported by (3) laboratory findings of elevated lactate and a decrease in the oxygen saturation of a mixed venous blood [11].

The patients included in this study were managed according to local standard protocols and current guidelines. The indication of a MCS implantation and choice of the best suitable MCS for each individual patient is based on the decision of a “shock team” consisting of an ICU physician, interventional cardiologist, and a heart failure specialist.

When a clinically significant arrhythmia appears, attempts are made to treat all possible underlying causes, including revascularization in coronary artery disease. As a next step, patients are placed on appropriate antiarrhythmic drugs. Furthermore, other measures suggested in the guidelines such as deep sedation, antitachycardic pacing, or stellate ganglion blockade are considered. The indication for a catheter ablation is based on a discussion between the shock team and the interventional electrophysiologists. The course of an ablation procedure then follows the valid guidelines according to the type of arrhythmia.

After discharge, CS patients are followed-up routinely at the outpatient clinic. For this analysis, a 30-day long follow-up period was chosen to reduce the number of patients lost, and to focus on the effect of the single catheter ablation intervention.

The study was performed according to the principles of good clinical practice and in compliance with the Declaration of Helsinki. The analysis of the registry was approved by the local ethical committee (approval No. 149/24 S-IV AP). The complete dataset was accessed for research purposes on the 01/11/2024. Authors had access to medical reports including information that could identify individual participants during or after data collection. Patient’s informed consent was waived given the retrospective and observational character.

The continuous variables are expressed as medians with interquartile range. The categorical variables are expressed as percentages.

## Results

### Ventricular tachycardias

We identified 9 CS patients (8 males, median age 69 years [IQR 60;74]) ablated for a refractory VT during the study period. Upon admission, 8 patients had a history of heart failure with reduced ejection fraction and 7 had a previously known coronary artery disease. An ICD had already been implanted in 5 patients. Three patients were admitted during the ongoing refractory cardiac arrest and the MCS implantation was performed as an extracorporeal cardiopulmonary resuscitation (ECPR) procedure. The median ejection fraction at the time of catheter ablation was 15% (IQR 10%; 20%) and the NT-proBNP value 6700 ng/l (IQR 4200; 12 100). Impella CP was used in 6 patients, 2 patients were on veno-arterial extracorporeal oxygenation (VA ECMO), and one patient was completely supported by the combination of VA ECMO and Impella CP for the left ventricle unloading (ECPELLA). The summary of patient’s characteristics is listed in Table 1.

**Table 1:** Patient’s medical history and characteristics.

Patient No.	Group	Sex	Age (years)	Height (cm)	Weight (kg)	Medical history	Cardiomyopathy aetiology	LV EF (%)	NT-proBNP (ng/l)	Antiarrhythmic drugs	MCS type
**01**	VT	M	60	180	106	ECPR, AH, DM, HFrEF, CAD, ICD	Ischemic	10%	16 054	Amiodarone Trimecaine	ECPELLA
**02**	VT	M	75	175	77	AH, HFrEF, CAD, ICD	Ischemic	15%	9 408	Amiodarone	Impella CP
**03**	VT	M	71	178	90	AH, DM, HFrEF, CAD	Ischemic	24%	25 781	Amiodarone, Trimecaine	VA ECMO
**04**	VT	F	74	168	80	HFrEF, MiR	Non-ischemic	10%	2 191	Amiodarone, Trimecaine	Impella CP
**05**	VT	M	50	180	104	AH, HFrEF, AoProst	Non-ischemic	10%	5 830	Amiodarone, Trimecaine	Impella CP
**06**	VT	M	36	180	100	ECPR, AH, CAD	Ischemic	20%	6 671	Amiodarone, Trimecaine	VA ECMO
**07**	VT	M	78	168	64	DM, STR, HFrEF, CAD, ICD, AoProst	Ischemic	30%	4 204	Amiodarone	Impella CP
**08**	VT	M	66	185	100	ECPR, DM, HFrEF, CAD, ICD	Ischemic	15%	2 608	Amiodarone	Impella CP
**09**	VT	M	67	173	83	AH, DM, CAD, HFrEF, ICD	Ischemic	20%	12 111	Amiodarone	Impella CP
**10**	SVT	M	85	180	85	AH, HFrEF, AoS	Non-ischemic	15%	5 999	Amiodarone	Impella CP
**11**	SVT	M	71	190	90	AH, DM, STR, HFrEF, CAD	Ischemic	25%	844	Amiodarone	Impella 5.5
**12**	SVT	M	53	175	88	STR, HFrEF, PulmR	Non-ischemic	30%	32 183	Digoxin	VA ECMO
**13**	SVT	F	75	167	73	AH, MiR	Non-ischemic	40%	5 782	Amiodarone	Impella CP

**Abbreviations:** AH – arterial hypertension, AoProst – aortic valve prosthesis (implanted), AoS – aortic valve stenosis (significant), CAD – coronary artery disease, DM – diabetes mellitus, ECPR – extracorporeal cardiopulmonary resuscitation, EF – ejection fraction, F – female, HFrEF - heart failure with reduced ejection fraction, ICD – implantable cardioverter defibrillator, M – male, MiR – mitral regurgitation (significant), PulmR – pulmonary regurgitation (significant), STR – stroke, SVT – supraventricular tachycardia, VT – ventricular tachycardia.

Clinical arrhythmia was recognized as a monomorphic VT in 6 patients, 1 patient had episodes of ventricular fibrillation triggered by monomorphic premature ventricular beats (PVCs), 1 patient was finally diagnosed with bundle branch re-entry VT, and 1 patient was ablated for polymorphic VTs. Before the ablation, all patients were on an antiarrhythmic treatment which included amiodarone.

The ablation procedure was performed on median day 2 (IQR 1; 4) of MCS. The median procedure time was 140 minutes (IQR 125; 180) and the median time of radiofrequency energy application was 34 minutes (IQR 26; 43). Clinical arrhythmia was observed in 5 patients during the ablation. Interestingly, despite the use of MCS, the arrhythmia presence was poorly hemodynamically tolerated in 2 patients (severe pulmonary oedema in 1 patient on the VA ECMO and hypotension in 1 patient on Impella CP). In two patients we failed to induce tachycardia, and in two other patients the tachycardia induction was not even performed because of their overall poor condition. In those cases, the ablation was based solely on the substrate mapping. The location of the ablation lesions, together with other details, is summarized in Table 2.

**Table 2:** Patients ablated for ventricular tachycardias—MCS and ablation details and outcomes.

Patient No.	MCS type	Successful weaning	Days on MCS	MCS complications	Arrhythmia type	Ablation approach	VT induction attempts	VT present during ablation	Ablated region	Ablation complications	VT recurrence	Death in 30 days
**01**	ECPELLA	No (LVAD implanted)	8	Bleeding	Monomorphic VT	Transsept.	Yes	No	Posterior, inferior LV	None	Yes	No
**02**	Impella CP	Yes	3	Haemolysis, renal failure	Monomorphic VT	Transsept.	Yes	No	LV apex	None	Yes	Yes
**03**	VA ECMO	Yes	9	None	PVC triggered V. fibrillation	Transsept.	No	Yes (PVC)	Infero-apical LV	Heart block	Yes	No
**04**	Impella CP	Yes	1	None	Bundle branch re-entry VT	Fem. vein	Yes	Yes	RV – RBB	None	No	No
**05**	Impella CP	Yes	6	Haemolysis	Monomorphic VT	Fem. art.	No	Yes	LVOT	None	No	No
**06**	VA ECMO	Yes	5	AV fistula	Polymorphic VT	Fem. art.	No	Yes	Inferior LV	None	Yes	No
**07**	Impella CP	Yes	1	Bleeding	Monomorphic VT	Transsept.	No	No	Posterior, inferior LV	None	No	No
**08**	Impella CP	No	10	None	Monomorphic VT	Transsept.	No	Yes	Anterior LV & apex	None	Yes	Yes
**09**	Impella CP	Yes	1	Acute limb ischemia	Monomorphic VT	Transsept.	No	No	LV apex, RV – RBB	None	No	Yes

**Abbreviations:** Fem. art. – femoral artery, Fem. vein – femoral vein, LV – left ventricle, LVOT – left ventricle outflow tract, MCS – mechanical circulatory support, No. – number, PVC – premature ventricular beat, RBB – right bundle branch, RV – right ventricle, Transsept. – transseptal, V. fibrillation – ventricular fibrillation, VT – ventricular tachycardia.

After the catheter ablation, 7/9 patients (78%) were successfully weaned off the MCS. The median total length of the MCS duration was 5 (IQR 1; 8) days. The one patient on ECPELLA who did not show signs of cardiac recovery was switched directly to a left ventricle assist device (LVAD) on the 8^th^ day post admission. Overall, 3 of the 9 patients who underwent ablation on MCS for refractory arrhythmia (33%) died during the first 30 days. One patient was consensually indicated to palliative care due to an overall poor state with multiorgan failure and died soon after the Impella weaning. Two other patients died from end-stage heart failure and multiorgan dysfunction several days after MCS removal. Ventricular arrhythmias recurred in 5/9 patients (56%). Two of them were those who subsequently died and one was the patient who underwent an early LVAD implantation as mentioned above. Post-ablation arrhythmias in the remaining two patients were successfully managed pharmacologically.

Clinically significant complications of MCS were reported in 6/9 patients (67%). Two patients had serious bleeding and required blood transfusions. Another two patients developed haemolysis leading to their renal function worsening while being on the ImpellaCP. The removal of the ImpellaCP using the Manta vascular closing device (Teleflex Inc., Morrisville, USA) in one patient with previously known peripheral artery disease was complicated by the acute limb ischemia due to the external iliac artery occlusion. This was solved by an acute surgical thrombectomy and endarterectomy, together with Manta device removal. One patient, after the emergent VA ECMO implantation during the ECPR, developed a significant arterio-venous fistula in the groin which required surgical ligation. The ablation procedure was complicated in only one patient by the transient complete heart block that required temporary ventricle pacing.

### Supraventricular tachycardias

Overall, 4 CS patients were ablated for SVT while being on a MCS (3 males, median age 73 years [IQR 67; 78]). One patient was on a VA ECMO, 2 patients on Impella CP, and one on Impella 5.5. Three patients had a history of heart failure with reduced ejection fraction. One patient had a known heart failure with preserved ejection fraction. Significant coronary artery disease was found in only one patient. In all four patients, there were repeated efforts to manage the arrhythmias pharmacologically before indicating the ablation procedure.

AF with rapid ventricular response was an indication for catheter ablation in 3 of the patients. All those patients were treated by a non-selective AV node ablation. Prior to the procedure, 2 patients had had a cardiac resynchronization therapy (CRT) device implanted. In one patient, conduction system pacing was used instead. The final patient underwent a radiofrequency ablation of a focal atrial tachycardia originating from the region of the left atrium above the coronary sinus. The ablation procedure in this patient lead to profound bradycardia with the eventual need for a pacemaker implantation. Besides that, there were no other reported complications of the ablation procedure. Pacemaker implantation was complicated in one patient by delayed bleeding, which required surgical revision. No infectious complications related to leads and pacemakers were reported during the 30-day follow-up.

On average, the ablation procedure was performed on median day 7 (IQR 4,75; 7,5) of MCS insertion. The median procedure time was 45 (IQR 44; 71) minutes. All procedures were successful, and there was no arrhythmia recurrence documented during the follow-up. All patients were successfully weaned off the MCS after the ablation. The median duration of MCS support was 13 days (IQR 8,5; 20). No patient died during the 30-day period after the ablation. One patient on Impella CP suffered from considerable haemolysis. No other MCS complications were reported. The data is summarized in Table 3.

**Table 3:** Patients ablated for supraventricular tachycardias—MCS and ablation details and outcomes.

Patient No.	MCS type	Successful weaning	Days on MCS	MCS complications	Arrhythmia type	Ablation approach	Ablated region	Ablation complications	Implanted device	SVT recurrence	Death in 30 days
**10**	Impella CP	Yes	9	Haemolysis	Focal SVT	Transsept.	Inferior LA, coronary sinus	Bradycardia, pacemaker implantation	Dual lead pacemaker	No	No
**11**	Impella 5.5	Yes	29	None	Atrial fibrillation	Fem. vein	AV node	None	CRT-D	No	No
**12**	VA ECMO	Yes	17	None	Atrial fibrillation	Fem. Vein	AV node	None	CRT-P (without atrial lead)	No	No
**13**	Impella CP	Yes	7	None	Atrial fibrillation	Fem. vein	AV node	None	Dual lead pacemaker (CSP)	No	No

**Abbreviations:** AV – atrio-ventricular, CSP – conduction system pacing, Fem. art. – femoral artery, Fem. vein – femoral vein, LA – left atrium, MCS – mechanical circulatory support, No. – number, SVT – supraventricular tachycardia.

## Discussion

This retrospective analysis illustrates the valuable role and feasibility of a catheter ablation procedure in the complex treatment of patients with CS and recurrent heart rhythm disorders.

According to the current guidelines, the implantation of an MCS should be considered in selected patients with CS when urgent hemodynamic stabilization is needed despite ongoing medical therapies [10,12]. The concrete device selection varies and depends on several factors including local availability and expertise as there is a lack of data for direct comparison [13]. Most of our patients (9/13) were treated with the Impella microaxial flow pump device. Recently, this approach has shown mortality benefit for patients with CS in the setting of a randomized trial [14].

Nowadays, the MCS are also being used more often during the catheter ablation, mainly for VTs, to improve the outcome [15]. According to the review by Kautzner et al., the MCS are used during the ablation in three different clinical scenarios: (1) pre-emptive use, (2) rescue mechanical support, and (3) bail-out ablation on MCS [16].

The **pre-emptive or prophylactic use** of MCS means that the patient is put on an MCS before the ablation because of the high risk of deterioration during the procedure. The MCS also potentially allows activation mapping of haemodynamically not tolerated tachycardias [16–18]. Interestingly, the prophylactic use of MCS in patients with structural heart disease undergoing catheter ablation was also associated with a significant reduction in postprocedural mortality or the need for heart transplantation [19].

The **rescue approach** refers to the situation of an acute deterioration during the ablation that requires urgent MCS implantation. In this setting, the VA ECMO is usually preferred. The prognosis of patients after the rescue MCS is generally poor, but this is mainly due to the serious underlying cause. However, data suggest that a modified rescue approach can be successfully used in intermediate-risk patients. Mascia et al. reported a case series of 10 patients undergoing VT ablation, in whom only vascular access with high-support leads was initially established, while the VA ECMO machine was prepared in the cath lab to enable rapid deployment if needed. Ultimately, only one patient required VA ECMO implantation, thereby avoiding unnecessary costs and risks [20].

The **bail-out ablation** is characterised by a situation when the catheter ablation is used to enable the MCS weaning. Patients analysed in this study therefore fit in this bail-out scenario. The data on bail-out ablation of ventricular arrhythmias in CS was reported by Ballout et al. in 2020. In this analysis of 21 consecutive patients, the aortic balloon pump was used most often. After the ablation, 17 subjects were successfully weaned off the MCS. In total, 6/21 (28,6%) patients died because of the persistent CS [21].

Interestingly, the published data on an SVT ablation in patients on MCS are scarce. In 2014, Cheruvu et al. reported a case of successful accessory pathway ablation in a patient with AV re-entrant tachycardia resulting in severe tachycardia-induced cardiomyopathy that required a VA ECMO implantation [22]. Later, Mantini et al. published a case series of 5 patients with CS who were ablated for SVT while being supported with different MCS [23]. To the best of our knowledge, other studies on this topic have not been published.

In our group, 3 out of 13 patients (23%) died during the 30-day period after the ablation. This is a lower mortality rate than usually reported for CS patients [13]. This can probably be explained by the selection bias. All patients were treated in the tertiary cardiac centre with the most advanced therapies available. It is also very probable that the most severe patients who deteriorated quickly were not included as they were not indicated for the ablation in spite of the presence of arrhythmia.

Despite the observed recurrence of the arrhythmia in 5/9 (56%) patients treated for VT, only two patients could not be successfully weaned off the MCS. This 7/9 (78%) successful weaning rate is comparable to the results published by Ballout el al. (17/21 ≈ 81%) [21].

In the SVT group, there was no recurrence of arrhythmia reported. This is not surprising as 3 out of 4 patients were treated with the AV node ablation – a procedure with a known higher success rate compared to a selective ablation for atrial fibrillation [24,25]. According to our experience, the “pace and ablate” approach seems to be a reasonable option for complicated patients with numerous comorbidities who suffer recurrent AF leading to a haemodynamic compromise.

It is well known that the use of an MCS is outweighed by the increased risk of complications. In our study, clinically significant complications due to the MCS implementation were reported in 7 of the 13 patients (54%). The complication rate of MCS found in the current literature varies substantially because of the non-uniformity of methodology and diverse complication criteria [26]. VA ECMO is usually connected with a higher complication rate than Impella [27]. To illustrate, the already mentioned randomized trials in CS patients reported a composite safety endpoint in 24% treated with Impella [14], and around 60% in patients treated with VA ECMO [28].

The ablation procedure itself seems to be relatively safe, even in this population of critically ill patients, according to both our results and the available data in literature [16,21,23]. The presence of an MCS; however, brings additional challenges to the operator. First, the availability of suitable cannulation sites may be limited. Also, navigating and controlling the catheters could be problematic in areas close to the ECMO cannula in the right atrium and especially in the left ventricle when Impella is used. The retrograde approach to the left ventricle may be another pitfall with Impella occupying the aortic ostium. Impella devices also cause electromagnetic interference that can significantly impair the performance of commonly used electroanatomic mapping systems, especially when higher flow (e.g., higher P-level) is used [29]. However, despite these limitations, all patients from our analysis treated with MCS underwent technically successful ablation.

### Limitations

We are aware that our analysis has numerous significant limitations. The most important seems to be its retrospective design. Despite the maximal effort and use of two independent registries (catheter ablation registry and MCS registry) we cannot rule out that some patients were missed. Moreover, it is probable that there were patients with CS and arrhythmias who were either not supported by an MCS or did not undergo an ablation procedure because of the severity of the illness or other comorbidities. Therefore, those patients could not be included. It is important to keep in mind that all patients were treated in a single, tertiary care cardiac centre with long-term experience in CS, MCS use, and catheter ablation. It is obvious that the small number of subjects prevents any advanced statistical analysis. The follow-up period of 30-days could be considered too short to cover all important events and give a comprehensive information about patients’ outcomes.

## Conclusion

Catheter ablation of refractory arrhythmias in CS patients treated by an MCS is a feasible approach to facilitate the MCS weaning process. Pace and ablate strategy seems reasonable in severe patients with refractory AF causing hemodynamic compromise when the chances to restore the sinus rhythm are low. The catheter ablation itself appears safe but complications of MCS are common. An individualized approach is necessary for the successful management of complicated CS patients.

## Supporting information

S1 Fig Graphical abstract. Catheter ablation in patients on mechanical circulatory supports for cardiogenic shock. Abbreviations: CS – cardiogenic shock, MCS – mechanical circulatory support, SVT – supraventricular tachycardia, VT – ventricular tachycardia **(Created in BioRender. Dusik,* ***M.*** *(2025)** https://BioRender.com/r94b354).
(TIF)

## References

[1] Arrigo M, Price S, Baran DA, Pöss J, Aissaoui N, Bayes-Genis A, et al. Optimising clinical trials in acute myocardial infarction complicated by cardiogenic shock: a statement from the 2020 Critical Care Clinical Trialists Workshop. Lancet Respir Med. 2021;9(10):1192–202. doi: 10.1016/S2213-2600(21)00172-7 34245691

[2] Sattler SM, Skibsbye L, Linz D, Lubberding AF, Tfelt-Hansen J, Jespersen T. Ventricular Arrhythmias in First Acute Myocardial Infarction: Epidemiology, Mechanisms, and Interventions in Large Animal Models. Front Cardiovasc Med. 2019;6:158. doi: 10.3389/fcvm.2019.00158 31750317 PMC6848060

[3] Aissaoui N, Puymirat E, Delmas C, Ortuno S, Durand E, Bataille V, et al. Trends in cardiogenic shock complicating acute myocardial infarction. Eur J Heart Fail. 2020;22(4):664–72. doi: 10.1002/ejhf.1750 32078218

[4] Cherbi M, Roubille F, Lamblin N, Bonello L, Leurent G, Levy B, et al. One-year outcomes in cardiogenic shock triggered by ventricular arrhythmia: An analysis of the FRENSHOCK multicenter prospective registry. Front Cardiovasc Med. 2023;10:1092904. doi: 10.3389/fcvm.2023.1092904 36776263 PMC9909601

[5] Tavazzi G, Dammassa V, Colombo CNJ, Arbustini E, Castelein T, Balik M, et al. Mechanical circulatory support in ventricular arrhythmias. Front Cardiovasc Med. 2022;9:987008. doi: 10.3389/fcvm.2022.987008 36304552 PMC9593033

[6] Volle K, Delmas C, Rollin A, Voglimacci-Stephanopoli Q, Mondoly P, Cariou E, et al. Successful Reversal of Severe Tachycardia-Induced Cardiomyopathy with Cardiogenic Shock by Urgent Rhythm or Rate Control: Only Rhythm and Rate Matter. J Clin Med. 2021;10(19):4504. doi: 10.3390/jcm10194504 34640519 PMC8509419

[7] Zeppenfeld K, Tfelt-Hansen J, de Riva M, Winkel BG, Behr ER, Blom NA, et al. 2022 ESC Guidelines for the management of patients with ventricular arrhythmias and the prevention of sudden cardiac death. Eur Heart J. 2022;43(40):3997–4126. doi: 10.1093/eurheartj/ehac262 36017572

[8] Van Gelder IC, Rienstra M, Bunting KV, Casado-Arroyo R, Caso V, Crijns HJGM, et al. 2024 ESC Guidelines for the management of atrial fibrillation developed in collaboration with the European Association for Cardio-Thoracic Surgery (EACTS). Eur Heart J. 2024;45(36):3314–414. doi: 10.1093/eurheartj/ehae176 39210723

[9] Brugada J, Katritsis DG, Arbelo E, Arribas F, Bax JJ, Blomström-Lundqvist C, et al. 2019 ESC Guidelines for the management of patients with supraventricular tachycardiaThe Task Force for the management of patients with supraventricular tachycardia of the European Society of Cardiology (ESC). Eur Heart J. 2020;41(5):655–720. doi: 10.1093/eurheartj/ehz467 31504425

[10] McDonagh TA, Metra M, Adamo M, Gardner RS, Baumbach A, Böhm M, et al. 2021 ESC Guidelines for the diagnosis and treatment of acute and chronic heart failure: Developed by the Task Force for the diagnosis and treatment of acute and chronic heart failure of the European Society of Cardiology (ESC) With the special contribution of the Heart Failure Association (HFA) of the ESC. Rev Esp Cardiol (Engl Ed). 2022;75(6):523. doi: 10.1016/j.rec.2022.05.005 35636830

[11] Vahdatpour C, Collins D, Goldberg S. Cardiogenic Shock. J Am Heart Assoc. 2019;8(8):e011991. doi: 10.1161/JAHA.119.011991 30947630 PMC6507212

[12] Heidenreich PA, Bozkurt B, Aguilar D, Allen LA, Byun JJ, Colvin MM, et al. 2022 AHA/ACC/HFSA Guideline for the Management of Heart Failure: Executive Summary: A Report of the American College of Cardiology/American Heart Association Joint Committee on Clinical Practice Guidelines. J Am Coll Cardiol. 2022;79(17):1757–80. doi: 10.1016/j.jacc.2021.12.011 35379504

[13] Laghlam D, Benghanem S, Ortuno S, Bouabdallaoui N, Manzo-Silberman S, Hamzaoui O, et al. Management of cardiogenic shock: a narrative review. Ann Intensive Care. 2024;14(1):45. doi: 10.1186/s13613-024-01260-y 38553663 PMC10980676

[14] Møller JE, Engstrøm T, Jensen LO, Eiskjær H, Mangner N, Polzin A, et al. Microaxial Flow Pump or Standard Care in Infarct-Related Cardiogenic Shock. N Engl J Med. 2024;390(15):1382–93. doi: 10.1056/NEJMoa2312572 38587239

[15] Mariani S, Napp LC, Lo Coco V, Delnoij TSR, Luermans JGLM, Ter Bekke RMA, et al. Mechanical circulatory support for life-threatening arrhythmia: A systematic review. Int J Cardiol. 2020;308:42–9. doi: 10.1016/j.ijcard.2020.03.045 32229050

[16] Kautzner J, Hašková J, Stojadinovič P, Peichl P, Wichterle D. Percutaneous mechanical support in catheter ablation of ventricular arrhythmias: hype or hope?. Europace. 2024;26(7):euae186. doi: 10.1093/europace/euae186 39028767 PMC11259133

[17] Baratto F, Pappalardo F, Oloriz T, Bisceglia C, Vergara P, Silberbauer J, et al. Extracorporeal Membrane Oxygenation for Hemodynamic Support of Ventricular Tachycardia Ablation. Circ Arrhythm Electrophysiol. 2016;9(12):e004492. doi: 10.1161/CIRCEP.116.004492 27932426

[18] Santangeli P, Rame JE, Birati EY, Marchlinski FE. Management of Ventricular Arrhythmias in Patients With Advanced Heart Failure. J Am Coll Cardiol. 2017;69(14):1842–60. doi: 10.1016/j.jacc.2017.01.047 28385314

[19] Muser D, Liang JJ, Castro SA, Hayashi T, Enriquez A, Troutman GS, et al. Outcomes with prophylactic use of percutaneous left ventricular assist devices in high-risk patients undergoing catheter ablation of scar-related ventricular tachycardia: A propensity-score matched analysis. Heart Rhythm. 2018;15(10):1500–6. doi: 10.1016/j.hrthm.2018.04.028 29753944

[20] Mascia G, Barca L, Sartori P, Bianco D, Della Bona R, Di Donna P, et al. Provisional Circulatory Support with Extracorporeal Membrane Oxygenation during Ventricular Tachycardia Ablation in Intermediate Risk Patients: A Case Series. J Clin Med. 2024;13(15):4477. doi: 10.3390/jcm13154477 39124744 PMC11312705

[21] Ballout JA, Wazni OM, Tarakji KG, Saliba WI, Kanj M, Diab M, et al. Catheter Ablation in Patients With Cardiogenic Shock and Refractory Ventricular Tachycardia. Circ Arrhythm Electrophysiol. 2020;13(5):e007669. doi: 10.1161/CIRCEP.119.007669 32281407 PMC7285871

[22] Cheruvu C, Walker B, Kuchar D, Subbiah RN. Successful ablation of incessant AV reentrant tachycardia in a patient on extracorporeal membrane oxygenation. Heart Lung Circ. 2014;23(1):e12-5. doi: 10.1016/j.hlc.2013.06.011 23921133

[23] Mantini N, Zipse M, Tompkins C, Varosy PD, Sauer WH, Nguyen DT. Ablation of atrial arrhythmias in patients with cardiogenic shock on mechanical circulatory support. HeartRhythm Case Rep. 2018;5(3):115–9. doi: 10.1016/j.hrcr.2018.11.008 30891405 PMC6404096

[24] Scheinman MM, Huang S. The 1998 NASPE prospective catheter ablation registry. Pacing Clin Electrophysiol. 2000;23(6):1020–8. doi: 10.1111/j.1540-8159.2000.tb00891.x 10879389

[25] Curtis AB, Kutalek SP, Prior M, Newhouse TT. Prevalence and characteristics of escape rhythms after radiofrequency ablation of the atrioventricular junction: results from the registry for AV junction ablation and pacing in atrial fibrillation. Ablate and Pace Trial Investigators. Am Heart J. 2000;139(1 Pt 1):122–5. doi: 10.1016/s0002-8703(00)90318-1 10618572

[26] Subramaniam AV, Barsness GW, Vallabhajosyula S, Vallabhajosyula S. Complications of Temporary Percutaneous Mechanical Circulatory Support for Cardiogenic Shock: An Appraisal of Contemporary Literature. Cardiol Ther. 2019;8(2):211–28. doi: 10.1007/s40119-019-00152-8 31646440 PMC6828896

[27] Ali JM, Abu-Omar Y. Complications associated with mechanical circulatory support. Ann Transl Med. 2020;8(13):835. doi: 10.21037/atm.2020.03.152 32793680 PMC7396259

[28] Ostadal P, Rokyta R, Karasek J, Kruger A, Vondrakova D, Janotka M, et al. Extracorporeal Membrane Oxygenation in the Therapy of Cardiogenic Shock: Results of the ECMO-CS Randomized Clinical Trial. Circulation. 2023;147(6):454–64. doi: 10.1161/CIRCULATIONAHA.122.062949 36335478

[29] Miller MA, Dukkipati SR, Koruth JS, d’Avila A, Reddy VY. How to perform ventricular tachycardia ablation with a percutaneous left ventricular assist device. Heart Rhythm. 2012;9(7):1168–76. doi: 10.1016/j.hrthm.2012.02.005 22322326

