# Supplementary figures and images for: Catheter ablation in patients on mechanical circulatory supports for cardiogenic shock
Source: PLoS One. 2025 Sep 15;20(9):e0332597. doi: 10.1371/journal.pone.0332597 (PMC12435639; doi:10.1371/journal.pone.0332597)

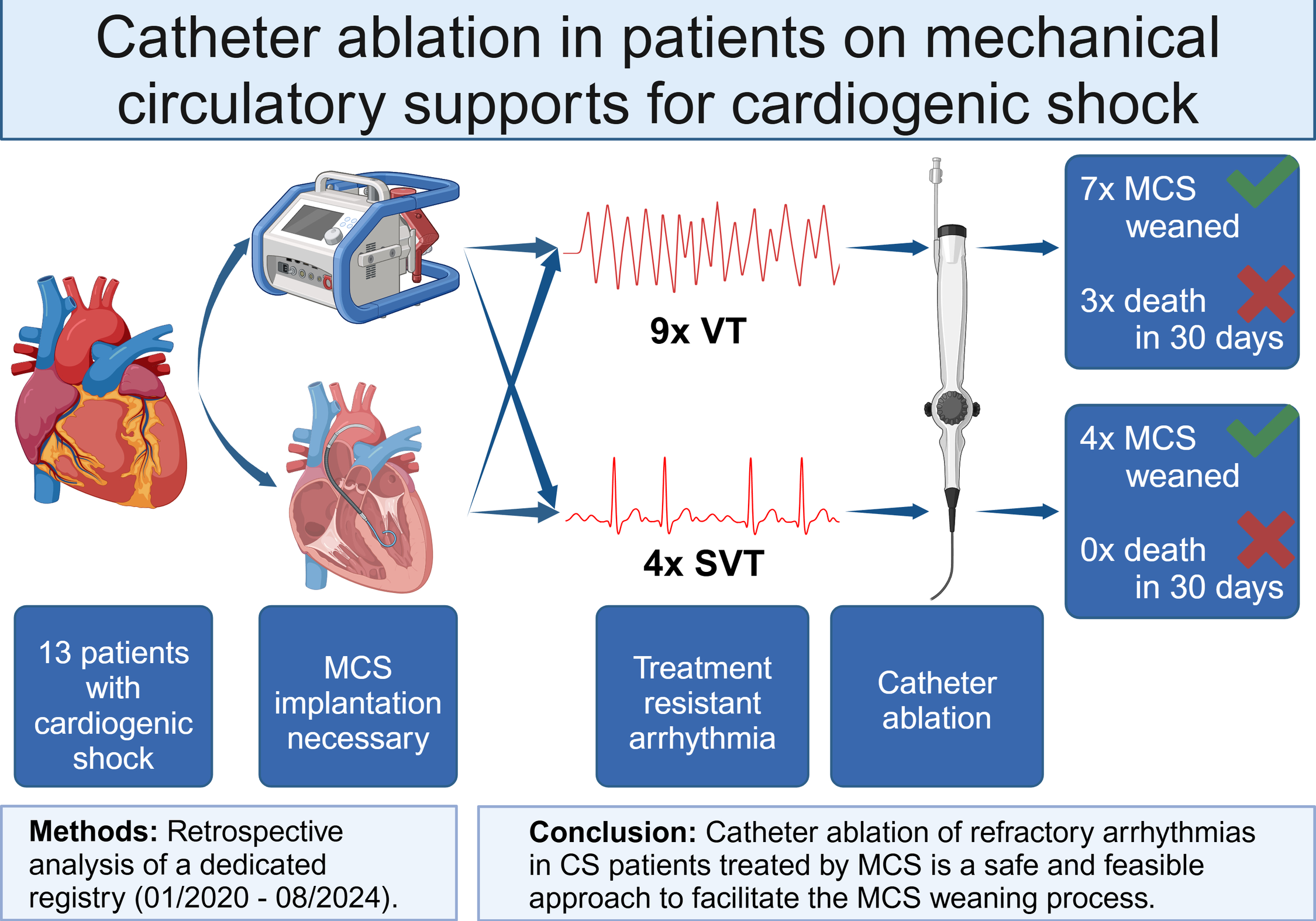

Supplement: S1 Fig — Catheter ablation in patients on mechanical circulatory supports for cardiogenic shock. Abbreviations: CS – cardiogenic shock, MCS – mechanical circulatory support, SVT – supraventricular tachycardia, VT – ventricular tachycardia (Created in BioRender. Dusik, M. (2025) https://BioRender.com/r94b354). (TIF) [file pone.0332597.s001.tif]
